# Supplementary material for: The spatial and temporal structure of neural activity across the fly brain
Source: Nat Commun. 2023 Sep 11;14:5572. doi: 10.1038/s41467-023-41261-2 (PMC10495430; doi:10.1038/s41467-023-41261-2)
Supplement: Supplementary file 1 — Supplementary Information [file 41467_2023_41261_MOESM1_ESM.pdf]

Expected number of neurons in the imaged volume. In order to estimate the expected number of neurons in our imaged volume, we refer to electron microscopy analyses of the fly brain, which reveal approximately 30,000 cells in the central brain (1). We imaged a parallelepiped-shaped volume spanning the dorsal third of the central brain. This volume should therefore contain roughly 10,000 cells. Half of these are Kenyon cells, which we could not see due to poor expression of our indicators, bringing our expected count to 5,000. Approximately the ventral third of the anterior and posterior faces of this volume are occluded by the cuticle due to the geometry of our microscope (see Fig. S1g). Given that the majority of cells are located along the perimeter of the brain, this occluded portion contains 1/3 of 1/2 (two of four sides) of the cells, bringing our expected count to 4167. Finally, in the ventral third of this volume, scattering prevented us from seeing every cell, meaning that our expected count should be reduced further (see Fig. S1h, blue).

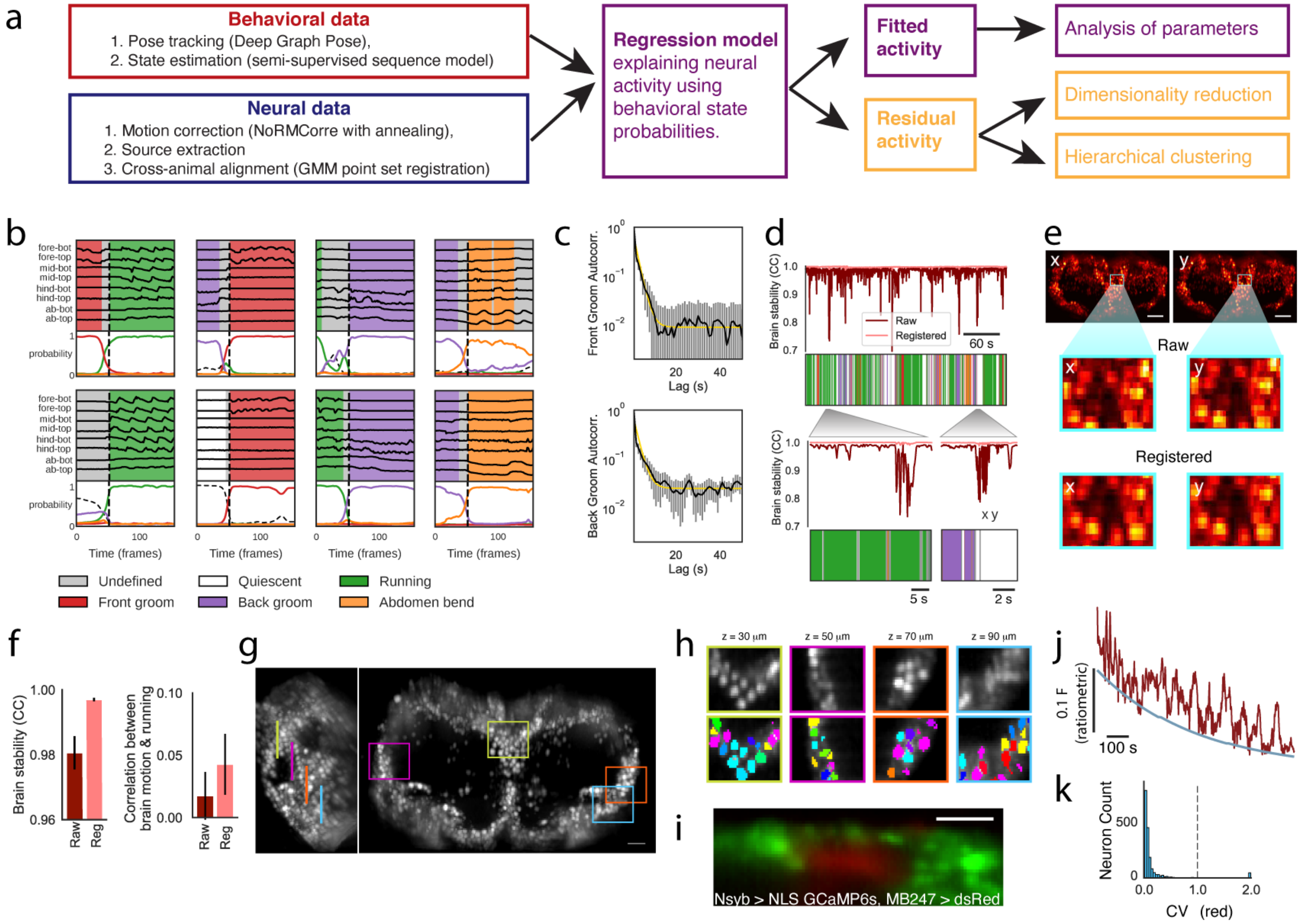

Figure S1. (a) Flow chart of analyses used in this work. (b) A semi-supervised sequence model (2) extracts a time series of discrete behavioral states from DGP points. Example trajectories of the 8 tracked points shown in black above, ordered from anterior to posterior (fb:front bottom, ft:front top, mb:middle bottom, mt:middle top, hb:hind bottom, ht:hind top, ab:abdomen bottom, at:abdomen top). Inferred probability of each behavioral state is shown below, showing a transition from running to back grooming. (c) The autocorrelation of front grooming (left, black) and back grooming (right, black) are best fit by a single exponential plus a constant offset, with time constants of 2s and 3s, respectively (gold). Error bars indicate $\pm$ SEM, N=18. (d) *Top,* motion of the brain volume before (dark red) and after (light red) registration, quantified as the correlation coefficient between red fluorescence and a single template image, with behavioral state shown below (same color code as in 'b'). The correlation between running and the raw and registered traces are -0.06 and -0.02, respectively. *Bottom,* magnified view of two epochs in which the brain moves substantially, during either running (left) or quiescence (right), with timepoints shown in 'e' indicated by 'x' and 'y'. (e) *Top,* snapshots of dsRed fluorescence from timepoints indicated by 'x' and 'y' markers in 'd', which are during and after an epoch of brain motion, respectively. *Middle,* magnified view of regions indicated by a cyan box above. *Bottom,* the same magnified regions after motion correction. (f) *Left,* average brain stability before and after registration, quantified as in 'd'. Bars denote standard error (N=18). *Right,* correlation between brain stability (quantified as in 'd') and running state probability, before and after registration. Bars denote standard error (N=18). (g) Maximum intensity projections over the dorsal/ventral and medial/lateral dimensions (right and left, respectively) of raw nuclear dsRed fluorescence from a dual color volume acquired in 0.1s *in vivo* from a representative fly. (h) Four z-slices at different depths, with x-y region color coded according to colored boxes in MIP image in 'g'. (i) Expression of NLS-GCaMP6s driven by Synaptobrevin (green), with Kenyon Cells labeled in red (MB247>dsRed) for a slice through the calyx in one hemisphere. Scale bar = 20 μm. (j) We define baseline ratiometric fluorescence (blue) as the best-fit exponential using least absolute deviation (LAD) regression applied to the derivative of ratiometric fluorescence. Raw ratiometric fluorescence shown in red. (k) To quantify residual motion and eliminate non-stationary cells, we compute the squared coefficient of variation from the red channel. Cells with values greater than 1 were rare and eliminated.

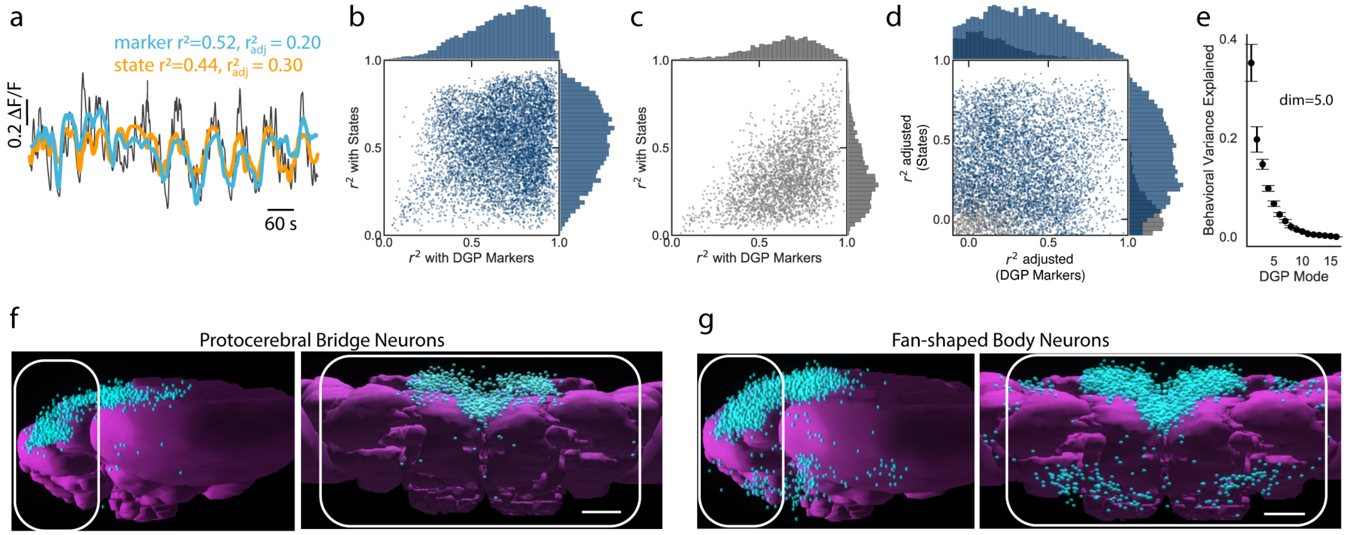

Figure S2. (a) $\Delta F/F$ from one cell (black), fit using either the principal components of DGP marker position (blue) or behavioral state probability inferred by the semi-supervised sequence model (gold) as regressors. The model using DGP marker position has x-y position for 8 tracked points, resulting in 16 regressors, whereas the model using state probabilities has 4 regressors (Running, front/back grooming, and abdomen bending). (b) Distribution of model fits (r^2) using DGP markers or behavioral state probabilities as regressors for all flies (N = 16), only showing cells whose fit was statistically significant using at least one of the models (temporally shifted regressor test, p<.05). (c) Same as 'b', showing only cells whose fit was not statistically significant for either model. (d) Same as 'a-c', but showing "r^2 beyond expected" rather than raw r^2. This is calculated as $(r^2 - r_0^2)/(1 - r_0^2)$, where r_0^2 is the average model fit for that cell using temporally shifted regressors (Cells with statistically significant fit for at least one model in blue, all other cells in gray). (e) Variance of DGP marker position explained by each principal component (DGP Mode). Average dimensionality of raw behavior as calculated by participation ratio is 5.0, consistent with the observation that the 'markers' model in 'a-d' performs similarly to the 'states' model (mean $\pm$ SEM, N=16). (F-G) Rendering of cell bodies from all neurons innervating either the Protocerebral Bridge (f) or the Fan-shaped Body (g) of the Central Complex. Renderings created using FlyCircuit (3). In each case, left and right show sagittal and transverse projections, respectively. White rectangles indicate approximate viewing window in our data, and scale bar = 50 μm.

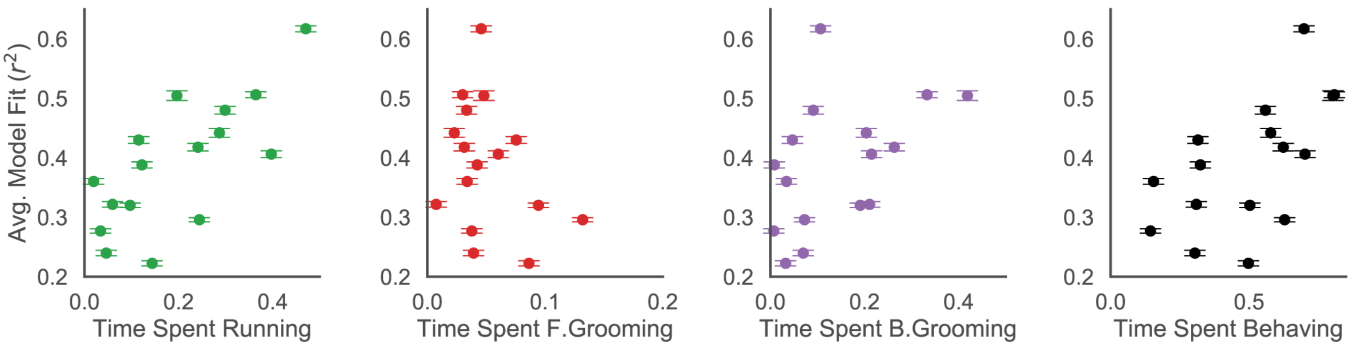

Figure S3. Fraction of variance explained by behavior regression model for each fly versus time spent running (green), front grooming (red), back grooming (purple), or the sum of all behaviors other than the quiescent and undefined states (black). Fraction of time spent running is most predictive of model fit (mean $\pm$ SEM, N=16).

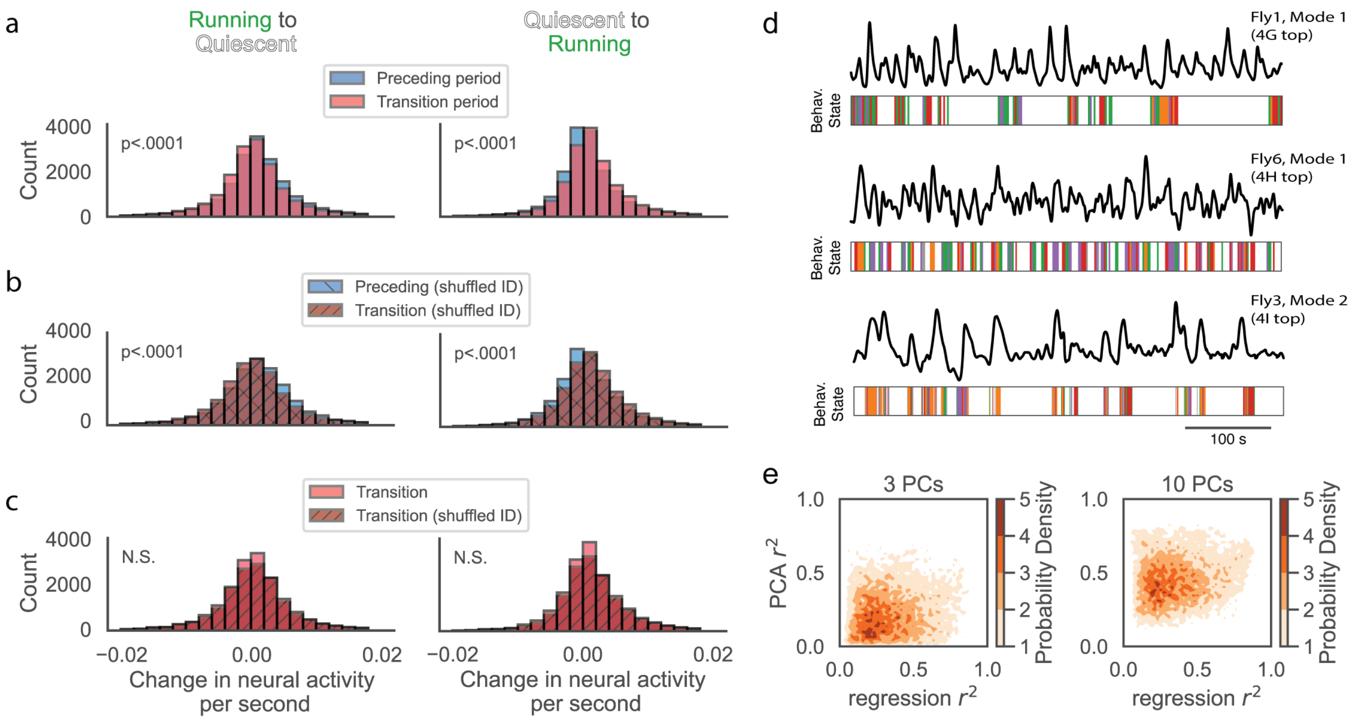

Figure S4. (a-c) Comparison of the change in neural activity at transitions from running to quiescence (left) and from quiescence to running (right). The "transition" period is the final half second before the transition, and the "preceding" period is the half second immediately before that. Change in activity per second for a given neuron is the average slope of activity over the time period, and the plotted quantity is the average for each neuron over all periods of a given type. (a) The average change in activity over all neurons is negative during transitions to quiescence (left) and positive during transitions to running (right); both shifts are small but significantly different than the values from the corresponding preceding periods (p<.0001, two-sample t-test). (b) Same as 'a' but shuffling cell identity before computing the slope of activity of each cell. For transitions to quiescence (left) and transitions to running (right), the effects seen in 'a' are maintained. (c) Comparison of transition periods in 'a' and 'b'. Means are not significantly different (two-sample t-test). (d) Timecourse of each of the three modes shown at the top of Figs. 4g-i, respectively, with ethograms shown below. Dynamics are in arbitrary units. (e) Variance explained by behavior regression model versus residual variance explained by first 3 PCs (left) or first 10 PCs (right) for all cells and all flies (N = 18). Variance accounted for by behavior (regression r^2) and variance explained by leading PCs (PCA r^2) show a statistically significant but negligible correlation. For 3 PCs, the Pearson's correlation coefficient was 0.06 and p < 0.001 and for 10 PCs, the correlation was 0.09 and p < 0.001. This implies that global and residual activity coexist in the same population of neurons. The statistical significance we observe in small correlations is unsurprising given the large number of data points $(n = 25,290)$.

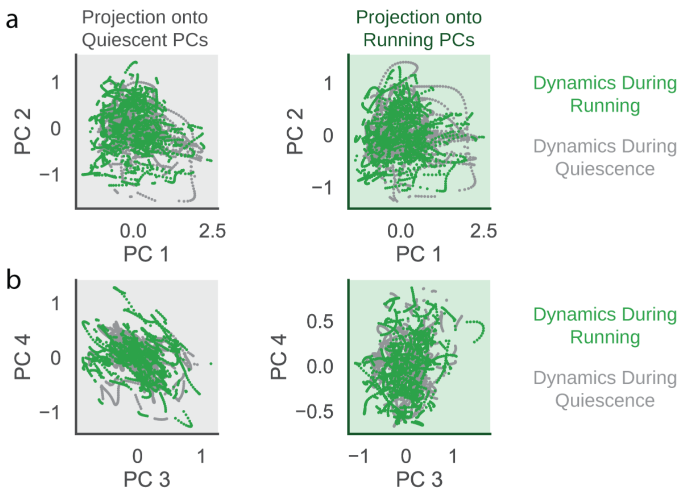

Figure S5. (a) Projections of the residual dynamics during running and quiescence onto the first two modes of the quiescent state (left) and the running state (right) for an example fly. (b) Same as 'a', for PCs 3 and 4.

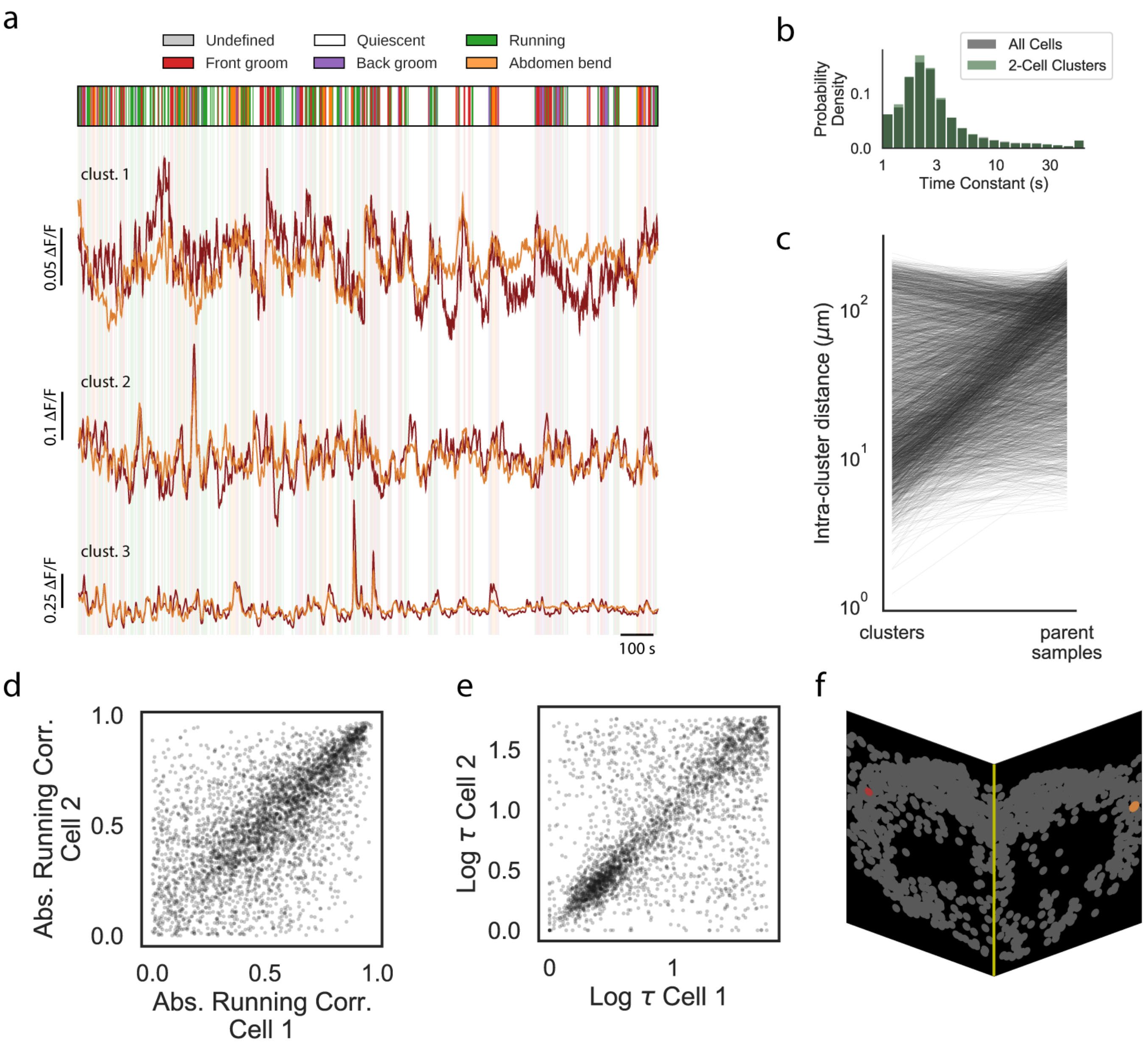

Figure S6. (a) Activity of cells from the three different clusters shown in Figure 6G-H before subtracting regression fit. (b) Distribution of behavior time constants for all cells (gray) and cells belonging to a significant two-cell cluster (green). (c) Intra-cluster distance of all 2-cell clusters versus the same quantity from samples of each cluster's parent. (d) Absolute value of correlation with running for each cell in a significant two-cell cluster versus absolute value of correlation with running for its partner cell. (e) Same as 'd', for log of behavior time constant. (f) To evaluate spatial organization of clusters, we calculate distance between member neurons after folding the volume along the midline (yellow).

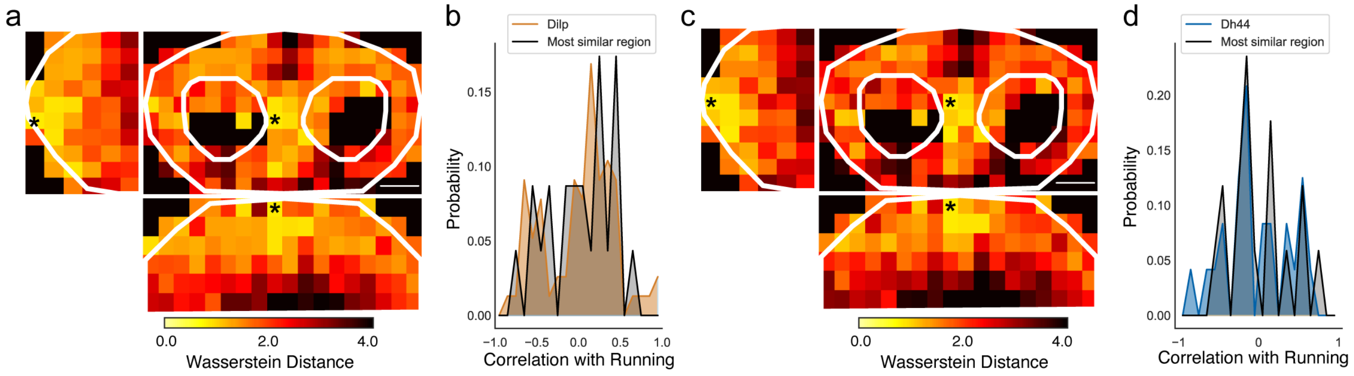

Figure S7. (a) Wasserstein distance between the distribution of running correlations among Dilp neurons versus local patches of neurons imaged with the panneuronal driver. The most similar region (patch with the smallest distance) is indicated with an asterisk and falls within the PI region. The contours of high cell body density are overlaid in white for reference. (b) Distribution of running correlations among Dilp neurons versus the optimal local patch of neurons indicated by an asterisk in 'a'. (c) Same as 'a', but for Dh44 neurons. (d) Same as 'b', but for Dh44 neurons.

Supplementary References

1. Scheffer L.K. et al. A connectome and analysis of the adult central brain. *Elife*, 9, September 2020.
2. Whiteway, M.R. et al. Semi-supervised sequence modeling for improved behavioral segmentation. *bioRxiv* June 2021.
3. Chiang, A.-S. et al. Three-dimensional reconstruction of brain-wide wiring networks in drosophila at single-cell resolution. *Curr. Biol.*, 21(1):1–11, January 2011.
