# Supplementary material for: The spatial and temporal structure of neural activity across the fly brain
Source: Nat Commun. 2023 Sep 11;14:5572. doi: 10.1038/s41467-023-41261-2 (PMC10495430; doi:10.1038/s41467-023-41261-2)
Supplement: Supplementary file 3 — Reporting Summary [file 41467_2023_41261_MOESM3_ESM.pdf]

Corresponding author(s): Evan S Schaffer
Last updated by author(s): 8/17/2023

Reporting Summary

Nature Portfolio wishes to improve the reproducibility of the work that we publish. This form provides structure for consistency and transparency in reporting. For further information on Nature Portfolio policies, see our Editorial Policies and the Editorial Policy Checklist.

Statistics

For all statistical analyses, confirm that the following items are present in the figure legend, table legend, main text, or Methods section.

n/a	Confirmed	
☐	☒	The exact sample size (*n*) for each experimental group/condition, given as a discrete number and unit of measurement
☒	☐	A statement on whether measurements were taken from distinct samples or whether the same sample was measured repeatedly
☐	☒	The statistical test(s) used AND whether they are one- or two-sided<br>*Only common tests should be described solely by name; describe more complex techniques in the Methods section.*
☒	☐	A description of all covariates tested
☒	☐	A description of any assumptions or corrections, such as tests of normality and adjustment for multiple comparisons
☐	☒	A full description of the statistical parameters including central tendency (e.g. means) or other basic estimates (e.g. regression coefficient) AND variation (e.g. standard deviation) or associated estimates of uncertainty (e.g. confidence intervals)
☐	☒	For null hypothesis testing, the test statistic (e.g. *F*, *t*, *r*) with confidence intervals, effect sizes, degrees of freedom and *P* value noted<br>*Give P values as exact values whenever suitable.*
☒	☐	For Bayesian analysis, information on the choice of priors and Markov chain Monte Carlo settings
☒	☐	For hierarchical and complex designs, identification of the appropriate level for tests and full reporting of outcomes
☐	☒	Estimates of effect sizes (e.g. Cohen's *d*, Pearson's *r*), indicating how they were calculated

Our web collection on statistics for biologists contains articles on many of the points above.

Software and code

Policy information about availability of computer code

Data collection	Data was collected using custom matlab software found in the below github repository interfacing with Andor acquisition system (andor.oxinst.com):<br>https://github.com/schafferEvan/VIP<br>(package versions are specified in the respective requirements.txt files of the repository)
Data analysis	Analyses were performed using custom python and matlab code that can be found in the following github repositiories (package versions are specified in the respective requirements.txt files in each repository):<br><br>https://github.com/schafferEvan/VIP<br>https://github.com/schafferEvan/flygenvectors<br>https://github.com/themattinthehatt/daart

For manuscripts utilizing custom algorithms or software that are central to the research but not yet described in published literature, software must be made available to editors and reviewers. We strongly encourage code deposition in a community repository (e.g. GitHub). See the Nature Portfolio guidelines for submitting code & software for further information.

Data

Policy information about availability of data

All manuscripts must include a data availability statement. This statement should provide the following information, where applicable:

- Accession codes, unique identifiers, or web links for publicly available datasets
- A description of any restrictions on data availability
- For clinical datasets or third party data, please ensure that the statement adheres to our policy

The data generated in this study have been deposited in a Figshare database at https://doi.org/10.6084/m9.figshare.23749074. Source data are provided with this paper. The datasets generated during the current study are publicly available in NWB format (https://www.nwb.org/). Our source data file is named "datasets_for_each_figure.xlsx".

Human research participants

Policy information about studies involving human research participants and Sex and Gender in Research.

Reporting on sex and gender	N/A
Population characteristics	N/A
Recruitment	N/A
Ethics oversight	N/A

Note that full information on the approval of the study protocol must also be provided in the manuscript.

Field-specific reporting

Please select the one below that is the best fit for your research. If you are not sure, read the appropriate sections before making your selection.

☒ Life sciences ☐ Behavioural & social sciences ☐ Ecological, evolutionary & environmental sciences

For a reference copy of the document with all sections, see nature.com/documents/nr-reporting-summary-flat.pdf

Life sciences study design

All studies must disclose on these points even when the disclosure is negative.

Sample size	This is an observational study. The analyses we conducted were not means tests. Power analyses for sample size were not relevant.
Data exclusions	All data with stable neural recordings and healthy fly behavior were included in this study.
Replication	All results were successfully replicated across multiple flies, and results from all analyzable datasets are reported.
Randomization	This was an observational study, there was nothing to be randomized.
Blinding	This was an observational study. Groups were not assigned and experimenters could not be blinded.

Reporting for specific materials, systems and methods

We require information from authors about some types of materials, experimental systems and methods used in many studies. Here, indicate whether each material, system or method listed is relevant to your study. If you are not sure if a list item applies to your research, read the appropriate section before selecting a response.

Materials & experimental systems

n/a	Involved in the study
☒	☐ Antibodies
☒	☐ Eukaryotic cell lines
☒	☐ Palaeontology and archaeology
☐	☒ Animals and other organisms
☒	☐ Clinical data
☒	☐ Dual use research of concern

Methods

n/a	Involved in the study
☒	☐ ChIP-seq
☒	☐ Flow cytometry
☒	☐ MRI-based neuroimaging

Animals and other research organisms

Policy information about studies involving animals; ARRIVE guidelines recommended for reporting animal research, and Sex and Gender in Research

Laboratory animals	5-7 day old female Drosophila melanogaster
Wild animals	no wild animals were used in the study
Reporting on sex	only female drosophila were used.
Field-collected samples	no field collected samples were used in the study.
Ethics oversight	this study did not require ethical approval.

Note that full information on the approval of the study protocol must also be provided in the manuscript.

March 2021
